# Randomized Dose-Ranging Controlled Trial of AQ-13, a Candidate Antimalarial, and Chloroquine in Healthy Volunteers

**DOI:** 10.1371/journal.pctr.0020006

**Published:** 2007-01-05

**Authors:** Fawaz Mzayek, Haiyan Deng, Frances J Mather, Elizabeth C Wasilevich, Huayin Liu, Christiane M Hadi, David H Chansolme, Holly A Murphy, Bekir H Melek, Alan N Tenaglia, David M Mushatt, Albert W Dreisbach, Juan J. L Lertora, Donald J Krogstad

**Affiliations:** 1 Center for Infectious Diseases, Tulane University Health Sciences Center, New Orleans, Louisiana, United States of America; 2 Department of Epidemiology, Tulane University Health Sciences Center, New Orleans, Louisiana, United States of America; 3 Department of Tropical Medicine, Tulane University Health Sciences Center, New Orleans, Louisiana, United States of America; 4 Department of Biostatistics, Tulane University Health Sciences Center, New Orleans, Louisiana, United States of America; 5 Department of Medicine, Tulane University Health Sciences Center, New Orleans, Louisiana, United States of America; 6 Department of Pharmacology, Tulane University Health Sciences Center, New Orleans, Louisiana, United States of America; 7 Tulane–Louisiana State University–Charity Hospital General Clinical Research Center, New Orleans, Louisiana, United States of America

## Abstract

**Objectives:**

To determine: (1) the pharmacokinetics and safety of an investigational aminoquinoline active against multidrug–resistant malaria parasites (AQ-13), including its effects on the QT interval, and (2) whether it has pharmacokinetic and safety profiles similar to chloroquine (CQ) in humans**.**

**Design:**

Phase I double-blind, randomized controlled trials to compare AQ-13 and CQ in healthy volunteers. Randomizations were performed at each step after completion of the previous dose.

**Setting:**

Tulane–Louisiana State University–Charity Hospital General Clinical Research Center in New Orleans.

**Participants:**

126 healthy adults 21–45 years of age.

**Interventions:**

10, 100, 300, 600, and 1,500 mg oral doses of CQ base in comparison with equivalent doses of AQ-13.

**Outcome Measures:**

Clinical and laboratory adverse events (AEs), pharmacokinetic parameters, and QT prolongation.

**Results:**

No hematologic, hepatic, renal, or other organ toxicity was observed with AQ-13 or CQ at any dose tested. Headache, lightheadedness/dizziness, and gastrointestinal (GI) tract–related symptoms were the most common AEs. Although symptoms were more frequent with AQ-13, the numbers of volunteers who experienced symptoms with AQ-13 and CQ were similar (for AQ-13 and CQ, respectively: headache, 17/63 and 10/63, *p* = 0.2; lightheadedness/dizziness, 11/63 and 8/63, *p* = 0.6; GI symptoms, 14/63 and 13/63; *p* = 0.9). Both AQ-13 and CQ exhibited linear pharmacokinetics. However, AQ-13 was cleared more rapidly than CQ (respectively, median oral clearance 14.0–14.7 l/h versus 9.5–11.3 l/h; *p* ≤ 0.03). QTc prolongation was greater with CQ than AQ-13 (CQ: mean increase of 28 ms; 95% confidence interval [CI], 18 to 38 ms, versus AQ-13: mean increase of 10 ms; 95% CI, 2 to 17 ms; *p* = 0.01). There were no arrhythmias or other cardiac AEs with either AQ-13 or CQ.

**Conclusions:**

These studies revealed minimal differences in toxicity between AQ-13 and CQ, and similar linear pharmacokinetics.

## Editorial Commentary

**Background:** Chloroquine (CQ) is a drug that has been widely used for over 40 years for the treatment and prevention of malaria. It is cheap, safe, and, except in areas where resistant malaria parasites exist, effective. However, the spread of resistant malaria parasites in most malarial regions of the world has meant that this drug, and many others, can no longer be relied upon to control disease. New drug candidates are therefore needed, and ideally should be cheap to produce as well as safe and effective. Some research groups are working on potential drug candidates from the aminoquinoline family of compounds, which includes chloroquine. One candidate, AQ-13 (aminoquinoline-13) has already been studied in animal and in vitro experiments, and seemed to be a good candidate for further testing in humans. Therefore, as the first stage in evaluating AQ-13 further, this group of researchers carried out a Phase I trial in healthy humans. The researchers specifically wanted to compare how often people given AQ-13, as compared to those given CQ, had side effects, and to find out how AQ-13 is handled in the body (i.e., how quickly the compound is taken into the bloodstream, gets broken down, and how it affects normal body functions). These sorts of studies do not tell researchers anything about the efficacy of the drug in treating malaria, but the results are absolutely essential before trials can be done that do test efficacy in people with malaria. 126 healthy volunteers were recruited into the study, and each received capsules containing a different dosage of either AQ-13 or CQ. Side effects data were collected for four weeks after the drugs were given.

**What this trial shows:** The most common side effects experienced by volunteers in the trial were headache, light-headedness, dizziness, and gastrointestinal symptoms such as nausea, vomiting, and diarrhea. Overall, the frequencies of such events were roughly similar among people receiving AQ-13 and those receiving CQ, but due to the small numbers of participants in the trial, it is not possible to say whether any observed differences in frequency of side effects between the two groups are meaningful or not. The data collected in this trial also showed that both AQ-13 and CQ were absorbed into the bloodstream in a similar way, but AQ-13 was absorbed more slowly than CQ. On ECG testing, both compounds increased the QT interval (part of the heart's electrical cycle, and used as a measure of heart function), particularly at high dosage levels, and volunteers given CQ experienced a greater increase in QT interval than those receiving AQ-13. No volunteers experienced any symptoms related to heart function. The researchers concluded that on the basis of these data, AQ-13 could proceed to further trials to evaluate the drug's efficacy in treating clinical malaria.

**Strengths and limitations:** The trial was appropriately designed as a randomized controlled Phase I study, allowing the researchers to assess safety and physiological outcomes after giving AQ-13 as compared to an existing and widely used drug, CQ. A key limitation inherent to such studies is the small number of participants studied. This means that the study cannot prove that AQ-13 is safe, or even as safe as CQ, but rather simply that the findings do not raise immediate safety concerns.

**Contribution to the evidence:** Data from animal experiments suggest that AQ-13 could potentially be a safe and effective antimalarial compound. The results reported here provide crucial safety data from the first study known to this research group that evaluates the effects of AQ-13 in humans.

## INTRODUCTION

Malaria is an overwhelmingly important public health problem with up to 3–4 billion cases and 3 million deaths each year [1,2]. In terms of malaria control and human health, chloroquine (CQ) was the most important antimalarial for more than 40 years because of its efficacy, safety, and affordability [3–5]. However, since the first reports of CQ-resistant Plasmodium falciparum in the 1960s [6,7] and the subsequent spread of CQ resistance across Southeast Asia, South America and sub-Saharan Africa [8], the single most important factor in the worldwide morbidity and mortality of malaria has been the increasing prevalence of CQ resistance in P. falciparum [9,10].

**Figure 1:**
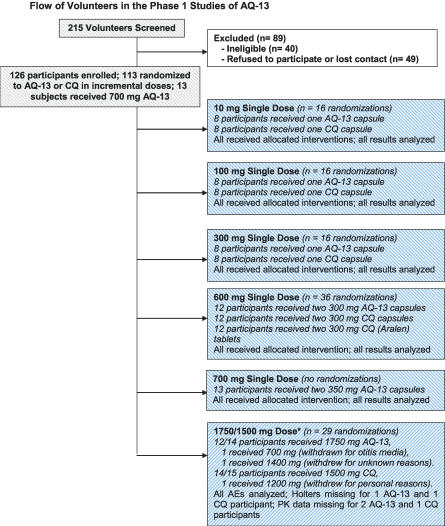
Phase 1 Randomized Clinical Trial of AQ-13 in Comparison with CQ Designed as a series of double-blind RCTs at incremental oral doses of 10, 100, 300, 600, and 1,500 mg, with a 700 mg adjustment dose after 600 mg to ensure similar bioavailability for AQ-13 and CQ at the 1,500/1,750 mg dose (based on the area under the curve, AUC_τ_, in h × μM, as in Figure 3). *AQ-13 dosages: 700 + 700 + 350 mg on days 1, 2, and 3, respectively; CQ dosages: 600 + 600 + 300 mg on days 1, 2, and 3, respectively.

Recent studies by ourselves and others have shown that aminoquinolines (AQs) with modified side chains are active against CQ-resistant P. falciparum in vitro [11–13]. Subsequently, we have shown that these AQs are as safe as CQ in mice and monkeys (Cogswell, et al., unpublished data), and are active in two monkey models of human malaria (P. cynomolgi in rhesus monkeys [14], which is a model of P. vivax infection in humans, and CQ-resistant P. falciparum in squirrel monkeys [15], which is a model of human CQ-resistant P. falciparum infection). The next step was to conduct a Phase I randomized clinical trial (RCT) to determine the safety and the pharmacokinetic behavior of the lead compound, AQ-13, in healthy volunteers (Figure 1).

**Figure 2:**
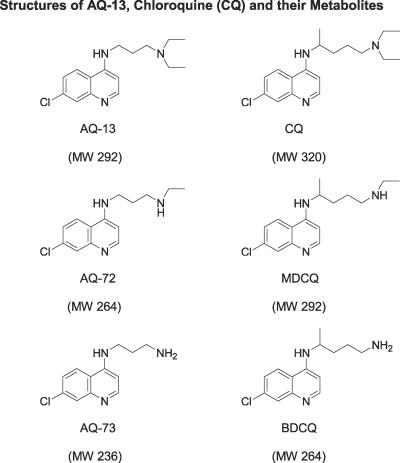
Structures of AQ-13, CQ, and Their Metabolites Two-dimensional structures are presented. Note that the AQ rings of AQ-13 and CQ are identical; the structural differences between AQ-13 and CQ are in their side chains: linear propyl side chain for AQ-13, branched isopentyl side chain for CQ. Therefore, the molecular weight (MW) of AQ-13 (292 Da) is 28 Da less than CQ (320 Da). Metabolism by *N-*dealkylation converts an ethyl group to a hydrogen (proton) at each step, resulting in stepwise MW differences of 28 Da.

**Figure 3:**
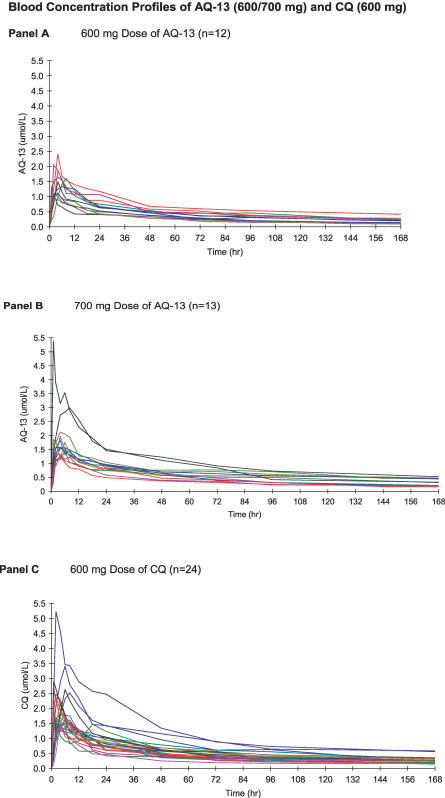
Pharmacokinetics of AQ-13 and CQ at Doses Equivalent to 600 and 700 mg CQ Base Charts of blood concentration data for individual volunteers during the first week (168 h) after: 600 mg dose of AQ-13 (A), 700 mg dose of AQ-13 (B), or 600 mg dose of CQ (C). Individual volunteers received single oral doses of 600 mg AQ-13 or CQ, or 700 mg AQ-13. Blood samples of 5 ml were then obtained at multiple points in time after drug administration (see Methods) and examined using a fluorescence HPLC assay for AQ-13, CQ, and their *N-*dealkylated metabolites [34]. Modeling was performed using the WinNonlin software (Pharsight).

### Selection of AQ-13 as the Lead Compound

Criteria for the selection of AQ-13 as the lead compound were: (1) in vitro activity against CQ-susceptible and –resistant *P. falciparum,* (2) activity in monkey models of human P. vivax and CQ-resistant P. falciparum infection, (3) safety, and (4) affordability. Based on these criteria, three AQs (AQ-13, AQ-21, and AQ-34) each could have been the initial lead compound. However, AQ-34, which has an isopropyl side chain, was dropped from consideration because the chiral center on its side chain resulted in two enantiomers. Because an additional (optical) purification would have been required to separate those enantiomers, the cost of pure AQ-34 would have been greater than that of AQ-13 or AQ-21. In addition, further studies would have been required to compare the activities and toxicities of the two enantiomers. Between AQ-13 and AQ-21 (which have linear propyl and ethyl side chains), AQ-13 (Figure 2) was chosen as the lead compound because it was more active in monkey models of human malaria (Cogswell, et al., unpublished data).

### Preclinical Studies of AQ-13 in Comparison with CQ

After AQ-13 had been selected as the initial lead compound, preclinical studies were performed to examine its toxicology and pharmacokinetics in animals in comparison with CQ [16,17]. Because those studies revealed no differences in toxicity between AQ-13 and CQ and similar pharmacokinetics, an Investigational New Drug Application was filed with the US Food and Drug Administration (IND 55,670) [18]. The rationale of that application was that an AQ active against CQ-resistant P. falciparum that was as safe and economical as CQ would be a major advance: because the few drugs effective against CQ-resistant P. falciparum are too expensive for use by the impoverished residents of malaria-endemic countries [19–21], because malaria parasites are already developing resistance to the expensive antimalarials now in use [22,23], and because there are unresolved concerns about the safety of the antimalarials now used to treat CQ-resistant P. falciparum [24,25].

Based on this information, the Phase I clinical trial reported here was performed as a series of RCTs to determine whether there were significant differences in toxicity (safety) or pharmacokinetics between AQ-13 and CQ in human volunteers.

## METHODS

### Participants

Healthy volunteers from 21 to 45 years of age were invited to participate in these studies. Exclusion criteria included pregnancy, breast-feeding, abnormal liver or kidney function tests, anemia (hemoglobin < 12 g/dl), chronic medications other than birth control pills, and an abnormal electrocardiogram (ECG) or Holter recording. Inpatient and outpatient studies were performed at the Tulane–Louisiana State University (LSU)–Charity Hospital General Clinical Research Center (GCRC) in New Orleans, Louisiana, United States.

There were two rationales for performing the Phase I studies of AQ-13 in the United States rather than in a malaria-endemic area: (1) ethical concerns of developing country colleagues and potential participants about drugs developed in the US are resolved most effectively by data indicating that the agent to be studied has been tested and shown to be safe in American volunteers, and (2) FDA regulatory staff required safety data from the US before considering studies of an investigational antimalarial in sub-Saharan Africa.

Informed consent was obtained from each volunteer before screening, based on a consent form approved by the Tulane Institutional Review Board. In addition, an independent Data Safety and Monitoring Board approved by National Institutes of Health, FDA, and the US Centers for Disease Control and Prevention reviewed the results for each dose with the principal investigator (DJK) and his colleagues before providing their permission to proceed to the next dose. The members of the Data Safety and Monitoring Board and their affiliations are listed below in the Acknowledgments section. Enrolment of volunteers began in August 1999 and follow-ups were completed in August 2005. Data entry was concluded in September 2005.

### Interventions

Participants were allocated randomly to receive the new candidate drug, AQ-13, or CQ. Sixteen volunteers were randomized to receive AQ-13 or CQ (eight each) at doses of 10, 100, or 300 mg base. At the 600 mg dose, 36 volunteers were randomized (12 each) to AQ-13 capsules, CQ capsules, or Sanofi-Winthrop CQ tablets (Aralen). AQ-13 was produced as the dihydrochloride, trihydrate salt under Good Manufacturing Practice (GMP) conditions by Starks Associates (Buffalo, New York, United States) and CQ as the phosphate salt by Sanofi-Winthrop (New York, New York, United States). Using this GMP material, University Pharmaceuticals (Baltimore, Maryland, United States) and SRI International (Menlo Park, California, United States) produced color-coded capsules containing equal molar doses of AQ-13 and CQ. Quality assurance and dissolution tests were performed by University Pharmaceuticals, SRI International and RTI (Research Triangle, North Carolina, United States) [18]. The third arm (commercially available CQ tablets) was included at the request of the FDA to determine whether there were differences between the CQ capsules prepared from GMP CQ phosphate (Sanofi-Winthrop) and commercially available CQ phosphate tablets (Aralen). Before the 1,500 mg therapeutic dose, 13 volunteers received a 700 mg adjustment dose of AQ-13 to compensate for the more rapid clearance of AQ-13. At the next stage of the Phase I study, 29 volunteers were randomized to receive either the standard therapeutic dose of 1,500 mg CQ base over 3 d or an equivalent 1,750 mg dose of AQ-13 (based on the adjustment dose).

#### Outpatient screening and inpatient admission.

To determine their eligibility, all volunteers had a complete physical exam, including an eye examination (visual acuity, visual fields, indirect ophthalmoscopy), were screened for hematologic and chemical abnormalities (complete blood count, chemistry panel including aspartate aminotransferase [AST], alanine aminotransferase [ALT], alkaline phosphatase, gamma-glutamyl transpeptidase [Gamma-GT], lactate dehydrogenase [LDH], bilirubin, creatinine, blood urea nitrogen [BUN], and fasting glucose), and for arrhythmias and other evidence of cardiac disease (physical exam, ECG, 24-hour Holter recording). Weight was measured by an electronic scale and height with a wall-mounted meter stick (Seca 216 Stadiometer, HealthCheck Systems, Brooklyn, New York, United States). Body mass index (BMI) was calculated using the formula: BMI = weight (kg)/height^2^ (m^2^).

Eligible volunteers were admitted as inpatients to the GCRC. Urine pregnancy testing was performed at the time of screening and again the evening before drug administration. Creatine kinase testing was also performed twice: at the time of screening and again on the evening of admission. Volunteers remained in the GCRC for 2.5–3.5 d depending on the AQ dose: 2.5 d for the 10, 100, 300, 600 mg and adjustment doses; 3.5 d for the 1,500/1,750 mg therapeutic dose.

#### AQ administration, and blood and urine samples for drug and metabolite levels.

Study drugs were administered in the GCRC on an empty stomach between 8 and 9 AM the morning after admission (after fasting for ≥ 10 h). For the first three doses, volunteers received single capsules containing 10, 100, or 300 mg CQ base or an equivalent molar amount of AQ-13 (9.1, 91.3, or 273.8 mg AQ-13 base, Figure 1). For the 600 mg dose and the 700 mg adjustment dose, volunteers received two 300 or 350 mg AQ-13 capsules (547.5 or 638.8 mg AQ-13 base) or two 300 mg CQ capsules, as a single morning dose. For the 1,500/1,750 mg therapeutic dose, volunteers received two 350 mg AQ-13 capsules or two 300 mg CQ capsules on days 1 and 2, and a single 350 mg AQ-13 or 300 mg CQ capsule on day 3 for doses of 1,750 mg AQ-13 (1,596.9 mg AQ-13 base) or 1,500 mg CQ.

Blood samples for drug and metabolite levels were obtained after the 600 mg dose, the 700 mg adjustment dose, and the 1,500/1,750 mg therapeutic dose, but not after the 10, 100, or 300 mg doses. Blood samples (5 ml) were drawn 0, 1, 2, 4, 6, 12, 18, 24, 48, 72, 96, and 120 hours after beginning AQ-13 or CQ administration, and twice weekly thereafter, up to 4 weeks.

#### Follow-up urine and blood samples.

In addition to the blood samples, twenty-four hour urine collections were obtained for 3 d after the 1,500/1,750 mg therapeutic dose to evaluate the urinary excretion of AQ-13, CQ, and their metabolites. Concentrations of AQ-13, CQ, and their metabolites were measured in whole blood and 24-hour urines with a fluorescence high-performance liquid chromatography (HPLC) assay using an Xterra RP18 analytical column with an elution buffer containing 60% borate (20 mM, pH 9.0) and 40% acetonitrile. Quantitation was based on the peak:area ratios for AQ-13, CQ, and their metabolites in relation to the internal standard [26].

#### Measurement of effects of AQ-13 and CQ on the QT interval.

After the 600 mg AQ-13 and CQ doses and the 700 mg (adjustment) AQ-13 dose, the QT interval was measured electronically from ECG recordings. The effects of the study drugs on the QT interval were assessed by comparing QT intervals before dosing with QT intervals 4 h after dosing, and at the 2 wk follow-up. After the 1,750 and 1,500 mg doses of AQ-13 and CQ, continuous 5 d Holter recordings were used to compare the effects of AQ-13 and CQ on the QT interval adjusted for a heart rate different from 60 beats per minute (QTc). Three 1-min recordings were examined from before dosing (baseline), from 4 and 5 h after each dose, and from 24 h after the last dose. QT intervals were measured manually and electronically (Rozinn Electronics, Glendale, New York, United States). Recordings obtained 48 h after the last dose, on the fifth day of Holter monitoring, were not used for analysis because they were of poor quality. Correction of the QT interval for heart rate (i.e., QTc) was performed using Bazett's formula [27].

#### Recording and reporting of adverse events.

Adverse events (AEs) were recorded in weekly diaries provided to each volunteer. The relatedness of these AEs to the study drugs was assessed by two physicians (FM, CH) based on temporal association and biological plausibility using five categories: definitely not, unlikely, possibly, probably, and definitely related. The AEs reported in this manuscript include all AEs assessed as possibly, probably, or definitely related. The one disagreement between these physicians was resolved by the principal investigator (DJK).

### Objectives

The basic and preclinical studies of AQ-13 and CQ [11–13,16–18] generated two hypotheses for the Phase I human studies: AQs structurally similar to CQ were likely to: (1) be safe in human volunteers, and (2) have side effects (AEs) and pharmacokinetics (blood levels and bioavailability) similar to those of CQ. Because AQ-13 was cleared more rapidly than CQ in the preclinical studies [17,18], the protocol for the Phase I human studies included a dose adjustment step after the 600 mg dose (Figure 1). Because the information available about the effects of CQ on the QT interval was limited [28,29], these studies used Holter recordings to compare the effects of CQ and AQ-13 on the QT interval.

Thus, the objectives of this Phase I trial were to determine: (1) the pharmacokinetics and safety of an investigational AQ active against resistant malaria parasites (AQ-13) [11–13], including its effects on the QTc interval, and (2) whether AQ-13 is likely to have pharmacokinetic and safety profiles similar to chloroquine (CQ) in humans. To address these questions, we performed a series of double-blind RCTs with incremental oral doses of AQ-13 and CQ equivalent to 10, 100, 300, 600, and 1,500 mg CQ base.

### Outcomes

Primary outcomes (endpoints) for the RCTs comparing incremental oral doses of AQ-13 and CQ included their pharmacokinetics, clinical and laboratory adverse events (AEs), and their effects on the QT interval. Pharmacokinetic parameters were calculated using the WinNonlin software (Pharsight, Mountain View, California, United States); they included: maximal drug concentration in the blood (C_max_), time from oral administration to C_max_ (T_max_), total area under the curve (AUC_τ_), terminal elimination half-life (t_1/2_), mean residence time (MRT), apparent oral clearance (Cl/F) and apparent volume of distribution (Vd/F). Clinical AEs were symptoms assessed as possibly, probably or definitely drug-related by the blinded physician reviewers that occurred within four weeks of drug administration. Laboratory AEs were abnormal hematologic or chemical test results identified within 4 d of drug administration or at the 2 or 4 wk follow-up. The effects of AQ-13 and CQ on the QT interval were defined in relation to the baseline QT interval, before AQ-13 or CQ administration.

Secondary outcomes (endpoints) evaluated were the pharmacokinetics of AQ-13 and CQ metabolites, pruritus after receiving AQ-13 or CQ, and ocular AEs [3,4,30–34].

### Sample Sizes

Sample sizes chosen for the lower doses (10, 100, and 300 mg) were eight in each group (AQ-13 and CQ) in order to detect one or more severe AEs in each dose–drug group with probabilities of 94% and 83%, assuming AE rates of 30% and 20%, respectively. Sample sizes chosen for the higher doses (600, 700, 1,500, and 1,750 mg) were 12 or 13 in each group in order to obtain a minimum of ten evaluable participants for pharmacokinetic studies within each dose–drug group and thus to detect one or more severe AEs in each dose–drug group with probabilities of 99%, 93%, and 72% based on AE rates of 30%, 20%, and 10%.

### Randomization

Volunteers who agreed to participate in the study, satisfied the inclusion and exclusion criteria, and completed their baseline studies were randomized to one of two or three treatments. Assignments of individuals to two treatments, A and B, were prepared by the study statistician by permuting blocks of four (A,A,B,B) and six (A,A,A,B,B,B) with a random number generator in a stepwise fashion—envelopes were prepared for each dose after the previous dose had been completed. The blocks of four and six were randomized so that block size was unknown to the investigators. For the comparison of three treatments, a similar procedure was performed for blocks of six (A,A,B,B,C,C) and nine (A,A,A,B,B,B,C,C,C). There was no stratification in this study. Assignments were then hand-delivered to the study pharmacist in opaque, sealed, numbered envelopes. On the morning(s) of drug administration, the study pharmacist opened those envelopes and dispensed the indicated drug (AQ-13 or CQ).

### Blinding

Neither the volunteers, the clinical or laboratory staff, nor the investigators knew which drugs the participants had received. Allocation codes and study drugs were controlled by the study pharmacist in the hospital pharmacy, which was outside the GCRC. Study drugs were dispensed the morning after admission after a phone call from the charge nurse indicating that a new volunteer had been admitted and was ready for drug administration. Interim data were reported to the Data Safety and Monitoring Board without breaking the code. Results and comparisons were reported for volunteers in two groups at the 10, 100, 300, and 1,500/1,750 mg doses (groups 1 and 2), and in three groups at the 600 mg dose (groups 1, 2, and 3). The staff, nurses, and investigators caring for volunteers in the GCRC and evaluating the relatedness of AEs to the study drugs were blinded; i.e., they did not know which drugs the participants had received.

### Statistical Methods

Drug concentration data for each participant were fitted to a noncompartmental pharmacokinetic (PK) model in order to estimate PK parameters using the WinNonlin 4.1 software (Pharsight). A noncompartmental model with extravascular input was chosen because it required fewer assumptions and because it better described the blood-concentration data [35]. Partial areas under the curve (partial AUCs) were calculated using the linear trapezoidal method up to the last blood concentration; total AUCs were then estimated by adding the extrapolated AUC from the last measurement to infinity [35]. Because the near-horizontal terminal slopes of the concentration–time data made the estimates of the extrapolated part of the area under the curve less reliable, oral clearance (Cl/F) was calculated from the formula (Cl/F = dose/AUC_obs_), where AUC_obs_ is the partial AUC based on the empirically observed data (for 4 wk). The multiple-dose model for the 1,500/1,750 mg therapeutic doses was derived using the nonparametric superposition method [35]. MRT was estimated using the statistical moments approach: (MRT = AUMC/AUC), where AUMC is the area under the first-moment concentration-time curve. Renal clearance (Cl_r_) was estimated from the means of the renal clearances for the three 24-h urine samples collected on days 1–3 after dosing, using the formula (Cl_r_ = X/pAUC) where X is the amount of the compound excreted in the urine, and pAUC is the partial blood AUC for the day of the urine sample. Because the terminal portions of the concentration–time curves for the metabolites were virtually flat in some cases, data for curves in which the extrapolated AUC exceeded 65% of the total AUC were not included in the analysis.

Quantitative data are presented as the mean ± standard deviation or as median and range, as appropriate. Fisher exact test or Pearson chi-square was used to compare the frequencies of the AEs reported for AQ-13 and CQ at each dose, and between African Americans and persons of European descent. Due to a lack of normality, the nonparametric Mann-Whitney U test was used to compare independent samples; the Wilcoxon or Friedman test (whichever was appropriate) was used to compare repeated measures of the QTc interval. All statistical tests were two-sided with an α (significance level) of 0.05. Analyses were performed using the SPSS 11.0 statistical software (SPSS, Chicago, Illinois, United States). All analyses were based on allocation by intent-to-treat. The small differences in the number of participants *(n)* across the tables in “Results” are due to the different numbers of missing data points for different outcomes.

This study had 70% power to detect a 50% difference in the frequency of AEs, assuming a 40% frequency of AEs in the control group (CQ). This power is based on combined dose groups, excluding the 10 mg dose. At the 600/700 mg dose level, this study had 80% power to detect a difference of 12 ms or greater in the mean change of the QTc interval from baseline. At the 1,750/1,500 mg doses, this study had 80% power to detect a difference of 15 ms or greater in the mean change of the QTc interval from baseline. All power calculations were performed using variances estimated from the study data.

## RESULTS

### Recruitment of Volunteers and Participant Flow

A total of 215 volunteers were screened to obtain 175 eligible participants (Figure 1). The remaining 40 volunteers were ineligible because they had abnormal chemistry or hematology lab results, abnormal ECGs or Holter recordings, or other health problems. Of the 175 eligible volunteers, 49 decided not to enroll or were lost because of scheduling conflicts or delays between screening and enrollment. Three volunteers withdrew after enrolment at the 1,500/1,750 mg dose; the participation of one volunteer was terminated by the supervising physician because of otitis media, and two volunteers dropped out for reasons unrelated to AEs after two doses (Figure 1). Of the 123 volunteers who received the planned doses of AQ-13 or CQ, 26 missed one or more of the eight follow-up visits, and 97 completed each of the follow-up visits. Available AE and Holter data for the three participants who withdrew were included in the analyses.

### Baseline Data and the Results of Randomization

Based on age, sex, race, weight, BMI, and the baseline QTc interval, there were no significant differences between volunteers randomized to AQ-13 versus CQ (Table 1). When baseline characteristics were compared at the different dose levels, there was a significant difference between the AQ-13 and CQ groups in mean weight (but not BMI) only at the 100 mg dose (unpublished data).

**Table 1:**
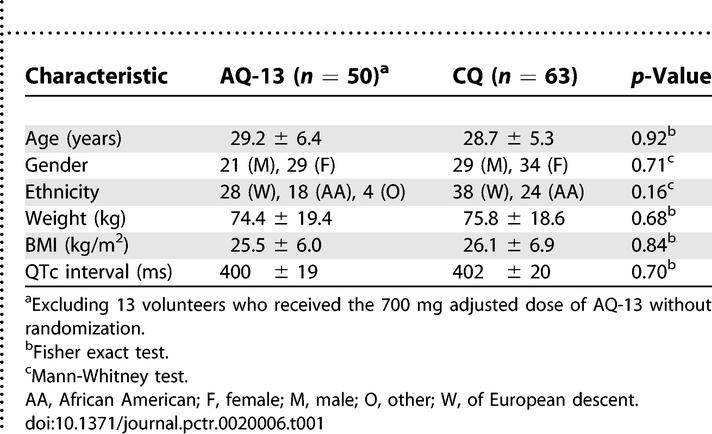
Randomization of Volunteers to AQ-13 and CQ: Baseline Characteristics

### Numbers Analyzed

All the 63 participants who received AQ-13 and the 63 who received CQ were analyzed for AEs, including those who withdrew before completing the intended dose. Holter data were available on 14 out of the 15 participants who received 1,500 mg CQ, and on 13 out of 14 participants who received 1,750 mg AQ-13, and were all included in the analysis.

### Outcomes and Estimation

#### Frequency of AEs.

The AEs reported most frequently were headache and lightheadedness/dizziness, which were distributed similarly among volunteers randomized to AQ-13 and CQ (Table 2). Headache was reported by 31 of 126 volunteers; 27 of those 31 reports were assessed as drug-related by the blinded physician reviewers. Of the 27 drug-related reports of headache, 17/63 (27%) were in volunteers who received AQ-13 and 10/63 (16%) were in volunteers who received CQ (*p* = 0.2). Lightheadedness/dizziness first appeared at the 300 mg dose level and was reported by 24/126 volunteers. Nineteen of those 24 reports were assessed as drug-related (11/63 and 8/63 for AQ-13 and CQ, respectively; *p* = 0.6). Gastrointestinal (GI) tract symptoms were the next most common AEs (nausea, diarrhea, vomiting, abdominal pain, loss of appetite), and first appeared at the 600 mg dose. The numbers of volunteers reporting one or more GI symptoms were similar in the two groups (AQ-13, 14/63; CQ, 13/63; *p* = 0.9). However, drug-related GI symptoms were reported more frequently by volunteers who received AQ-13 (28 reports) than volunteers who received CQ (20 reports), because GI symptoms were more clustered in volunteers treated with AQ-13. Other symptoms, such as mild, transient eye (blurred vision, difficulty focusing, floating objects) or ear symptoms (changes in hearing, ringing in the ears), mild skin rash (one volunteer had a sparse maculopapular erythematous eruption on the lower torso), and fatigue were infrequent and occurred at similar frequencies in both groups. CQ pruritus was not reported by any of the volunteers. Because of the small numbers of volunteers studied at each dose, no significant conclusions can be drawn from comparisons of AEs between drugs at the individual dose levels. There were no differences in the incidence of AEs between African American volunteers and those of European descent (*p* = 0.63).

**Table 2:**
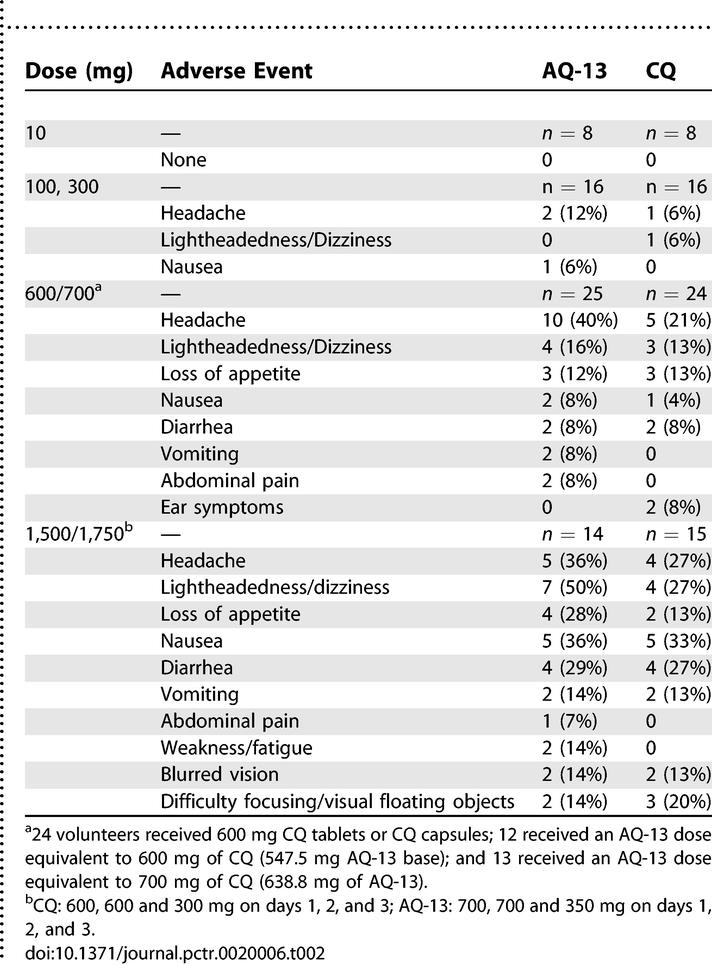
Frequency of Related Adverse Events by Drug and Dose

#### Post-dose clinical and laboratory follow-up.

There was no clinical evidence for end-organ toxicity after either AQ-13 or CQ during the daily inpatient examinations, or at the 2 or 4 wk outpatient follow-up. Specifically, there was no evidence for cardiac, dermatologic, hepatic, hematologic, ocular, or other organ toxicity.

Repeat laboratory testing 4 d after drug administration revealed no hematologic or chemical toxicities with either AQ-13 or CQ. At the 2 wk follow-up, two volunteers who received AQ-13 and two who received CQ had mildly abnormal liver function tests (AQ-13: one participant with a bilirubin of 1.5 mg/dl, another participant with an AST of 135 U/l, an ALT of 149, and an alkaline phosphatase of 146; CQ: one participant with a bilirubin of 1.7 and another with an ALT of 50). The two volunteers who received AQ-13 had received the 100 and 300 mg doses; the two volunteers who received CQ had both received the 1,500 mg dose. Follow-up test results were normal for all participants at the times of the 3 and 4 wk outpatient visits.

#### Pharmacokinetics of AQ-13 and CQ.

The 600 mg doses of AQ-13 and CQ were absorbed rapidly after oral administration (Figure 3). Blood levels of AQ-13 and CQ peaked at similar times (T_max_ 4.0 h [1.0–8.0 h] and 3.0 h [1.0–8.0 h] for AQ-13 and CQ), but had different maximal concentrations (C_max_ 1.4 μM [0.9–2.4 μM] and 1.8 μM [1.3–5.2 μM] for AQ-13 and CQ; *p* < 0.01), and the absorption of CQ was slightly more rapid than AQ-13 (Table 3). One hour after dosing, the CQ blood level was 72% of the CQ C_max_ versus 52% for AQ-13. AQ-13 had a shorter terminal elimination t_1/2_ than CQ (14.3 [6.2–39.3 d] versus 23.3 d [10.2–54.6 d]; *p* < 0.01), a shorter MRT (10.5 d [6.0–37.4 d] versus 24.7 d [12.4–49.8 d]; *p* < 0.01), a smaller AUC_τ_ (140.8 h × μM [63.4–351.9] versus 241.2 h × μM [179.8–432.4]; *p* < 0.01), and was cleared more rapidly (Cl/F = 14.7 l/h [7.0–31.1 l/h] versus 11.3 l/h [5.7–20.3 l/h]; *p* = 0.01) (Table 3). However, no PK differences were observed between the results obtained with the GMP CQ capsules and the standard CQ tablets available commercially (Aralen, *p* ≥ 0.15 for all PK parameters).

**Table 3:**
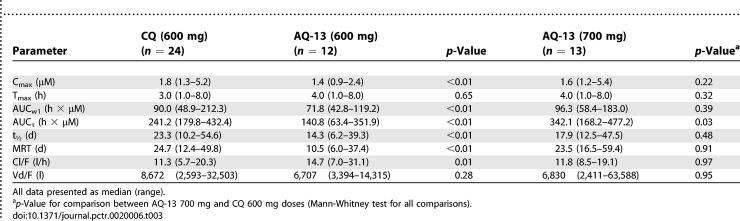
Comparative Pharmacokinetics of AQ-13 and CQ at 600 and 700 mg Doses

With the 700 mg adjustment dose of AQ-13, the lower C_max_ and the smaller AUC_τ_ of AQ-13 at the 600 mg dose (Figure 3A; Table 3) indicate that AQ-13 is less bioavailable than CQ, cleared more rapidly than CQ, or both. To compensate for the apparent lower bioavailability of AQ-13 and achieve similar systemic exposure (based on the AUC)—in order to compare the safety of AQ-13 and CQ—the AQ-13 dose was increased (adjusted) to 700 mg and compared with the 600 mg dose of CQ (Figure 3B and 3C). Because the major metabolite of AQ-13 (mono-*N*-dealkylated AQ-13) is not active against CQ-resistant parasites, this adjustment was based on the AUC_τ_ for the parent compound (AQ-13), and did not consider either of its metabolites. The 700 mg dose of AQ-13 was administered to 13 healthy volunteers using the same protocol. The 700 mg dose of AQ-13 produced a larger AUC_τ_ than 600 mg CQ, but a similar first-week partial AUC (AUC_w1_), and a similar mean C_max_ (Table 3). Based on these results, the 1,500 therapeutic dose of CQ was compared with 1,750 mg of AQ-13 in the last part of the study.

In the comparison of the 1,500 mg therapeutic dose of CQ with 1,750 mg AQ-13, the 1,750 mg AQ-13 dose produced a smaller AUC_τ_ than 1,500 mg CQ, although this difference was of borderline significance (*p* = 0.09; Table 4). The more clinically relevant AUC_w1_ and mean C_max_ tended to be lower in volunteers who received AQ-13 than in volunteers who received CQ, although these differences were not significant (*p* = 0.3). These results are consistent with the 600/700 mg dose because AQ-13 was cleared more rapidly than CQ (Cl/F = 14.0 l/h [6.8–20.3 l/h] and 9.5 l/h [5.4–20.6 l/h]; *p* = 0.03). However, the terminal elimination t_½_ and MRT were similar with AQ-13 and CQ (*p* = 0.87, *p* = 0.89; Table 4). With both AQ-13 and CQ, peak blood concentrations were achieved 3–4 h after the second dose (27–28 h after the first dose).

**Table 4:**
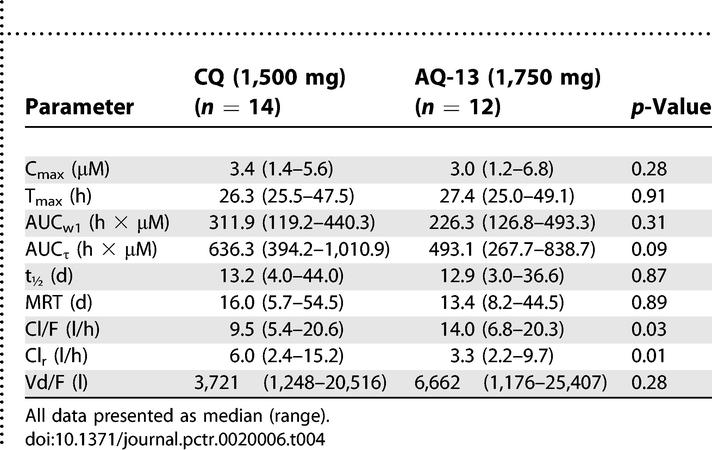
Comparative Pharmacokinetics of AQ-13 and CQ at 1,750 and 1,500 mg Doses

#### Pharmacokinetics of AQ-13 and CQ metabolites.

Mono-*N*-dealkylated AQ-13 and CQ (AQ-72 and MDCQ) are the major metabolites of AQ-13 and CQ [33]. Both AQ-72 and MDCQ appeared in the blood within 1 h after the oral administration of 600 or 700 mg of AQ-13 or 600 mg of CQ (Table 5), and were identified in all but two of 60 participants (one each with AQ-13 and CQ). Although the di*-*dealkylated metabolites of AQ-13 and CQ (AQ-73, BDCQ) were not detected in the blood, they were identified in urine collections from days 1–3 after dosing.

**Table 5:**
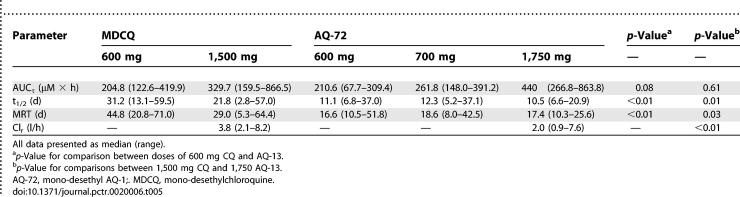
Comparative Pharmacokinetics of AQ-72 and MDCQ at Different Doses of AQ-13 and CQ (*median, range*)

The pharmacokinetics of AQ-72, the initial metabolite of AQ-13 (600 mg AQ-13), were similar to those of the parent drug (median MRT of 16.6 d [10.5–51.8 d] for AQ-72 versus 10.5 d [6.0–37.4 d] for AQ-13; *p* = 0.16; median terminal elimination t_1/2_ of 11.1 d [6.8–37.0 d] for AQ-72 versus 14.3 d [6.2–39.3 d] for AQ-13; *p* = 0.67). The median C_max_ of AQ-72 was 0.59 μM (0.25–0.76 μM) with a median T_max_ of 15 h (8.0–48.0 h). At the C_max_ of AQ-13, the AQ-72/AQ-13 ratio varied widely with a mean of 0.37, which increased rapidly thereafter and reached 1.0 3–6 d after drug administration.

In contrast, the pharmacokinetics of MDCQ, the initial metabolite of CQ (600 mg CQ), were different from those of CQ; MDCQ had a longer MRT and terminal t_1/2_ than CQ (median MRT of 44.8 d [20.8–71.0 d] versus 24.7 d [12.4–49.8 d]; *p* < 0.01; median terminal t_1/2_ of 31.2 d [13.1–59.5 d] for MDCQ versus 23.3 d [10.2–54.6 d] for CQ; *p* = 0.01). At the CQ C_max_, the MDCQ/CQ ratio was 0.26; it then increased slowly until it reached 1.0 2–3 wk after dosing. Despite the lower C_max_ of MDCQ, the AUC_τ_ values for MDCQ and CQ were similar (MDCQ 204.8 [122.6–419.9]; CQ 241.2 [179.8–432.4]; *p* = 0.32) because of the longer terminal elimination t_1/2_ with MDCQ.

In a comparison of AQ-72 and MDCQ (600 mg AQ-13/CQ), estimates of C_max_ were similar for AQ-72 and MDCQ (0.59 μM [0.25–0.76 μM] versus 0.54 μM [0.32–0.89 μM]; *p* = 0.26)*.* However, MDCQ had a longer median MRT than AQ-72 (44.8 d [20.8–71.0 d] versus 16.6 d [10.5–51.8 d]; *p* < 0.01), and a longer terminal elimination t_1/2_ (31.2 d [13.1–59.5 d] versus 11.1 d [6.8–37.0 d]; *p* < 0.01). Similar results for both AQ-72 and MDCQ were obtained at the 1,750/1,500 mg doses, except MRT was shorter with MDCQ at the 1,500 mg dose than at the 600 mg dose (Table 5).

The amounts of unchanged drug recovered from 24 hour urine collections on days 1–3 were 8.4% and 18.0% of the total oral doses of AQ-13 and CQ, respectively (443 μmol [304–645 μmol] and 829 μmol [530–1,202 μmol]). Although the AQ-72/AQ-13 and MDCQ/CQ ratios in urine were similar (23.6% and 22.7%), when comparing the ratios of the second metabolite to the parent drugs, the AQ-73/AQ-13 ratio was twice as large as the BDCQ/CQ ratio (5.2% and 2.6% for AQ-73 and BDCQ), consistent with more effective conversion of AQ-13 to its mono- and di-dealkylated metabolites, more rapid Cl_r_ of AQ-73 than BDCQ, or both. The Cl_r_ of AQ-13 was less than that of CQ (*p* = 0.01; Table 4). Similarly, the renal clearance of AQ-72 was less than that of MDCQ (*p* < 0.01; Table 5).

### Effects of AQ-13 and CQ on the QT Interval

#### Baseline QTc intervals.

Mean ± standard deviation (range) baseline QTc intervals were similar in the AQ-13 and CQ groups (Table 6). At the 600/700 mg dose level, QTc interval duration was 403 ± 17 ms (376–445 ms), and 406 ± 19 ms (369–448 ms) for AQ-13 and CQ (*p* = 0.65), while at the 1,500/1,750 mg dose level QTc was 397 ± 16 ms (373–421 ms) and 396 ± 21 ms (362–430 ms) for AQ-13 and CQ (*p* = 0.9). Likewise, there were no differences in the median baseline QTc intervals between males randomized to CQ versus AQ-13, or between females randomized to CQ versus AQ-13.

**Table 6:**
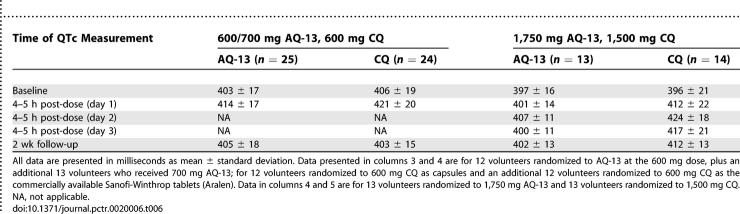
Effects of AQ-13 and CQ on the QTc Interval

#### Effects of AQ-13 and CQ on the QTc interval.

Both AQ-13 and CQ prolonged the QTc interval at doses of 600/700 and 1,500/1,750 mg. CQ produced greater prolongation of the QTc interval than AQ-13 (Table 6).

Four hours after drug administration, volunteers who received 600 mg CQ had a mean 16 ms (95% confidence interval [CI], 9 to 23 ms) increase in the QTc interval from baseline, in comparison to an 11 ms (95% CI,, 4 to 18 ms) increase after 600 or 700 mg AQ-13. When the data were analyzed by gender, significant increases in the QTc interval were observed only for females with both drugs (AQ-13, 18 ms increase [95% CI, 10 to 27 ms]; CQ, 22 ms increase [95% CI, 14 to 31 ms]). In contrast, mean QTc interval changes were not significant for males with either AQ-13 or CQ (AQ-13, 1 ms; CQ, 7 ms; *p* > 0.3 for both). Among the 49 male and female volunteers who received 600/700 mg AQ-13 or 600 mg CQ, two volunteers developed QTc intervals greater than 450 ms (467 ms and 457 ms). Both were female, both had received CQ; neither had any cardiac AEs.

**Figure 4:**
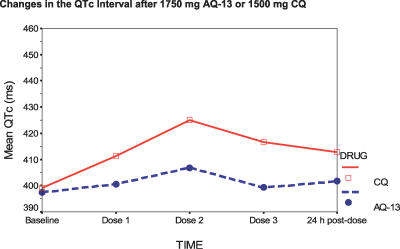
Changes in the QTc Interval after 1,750 mg AQ-13 or 1,500 mg CQ Changes in the QTc interval from baseline were determined using the Rozinn Electronics system software to evaluate the Holter recordings.

**Figure 5:**
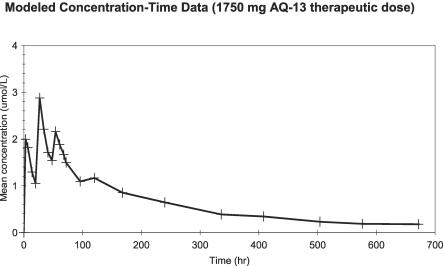
Modeled Concentration-Time Data (1,750 mg AQ-13 Therapeutic Dose) Individual volunteer*s* received daily oral doses of AQ-13 for 3 d (day 1, 700; day 2, 700; and day 3, 350 mg). Blood samples were then obtained, analyzed, and modeled (see Methods).

**Figure 6:**
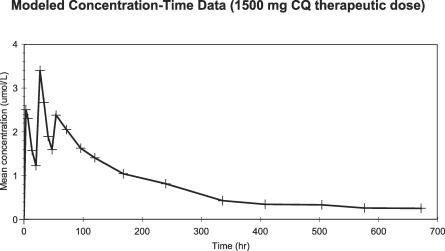
Modeled Concentration–Time Data (1,500 mg CQ Therapeutic Dose) Individual volunteers received daily oral doses of CQ for 3 d (day 1, 600; day 2, 600; and day 3, 300 mg). Blood samples were then obtained, analyzed and modeled (see Methods).

On the other hand, for the 1,750 mg AQ-13, 1,500 mg CQ dose, after the therapeutic dose, the effects of AQ-13 and CQ on the QTc interval were parallel to their blood levels—that is, QTc prolongation was greatest 4 h after the second dose on day 2, which was the time of the peak blood levels for both drugs (Figures 4–6). With AQ-13, the mean ± standard deviation QTc interval increased from 397 ± 16 ms at baseline to 407 ± 11 ms 4 h after the second dose (*p* = 0.025). With CQ, the mean QTc interval increased from 396 ± 21 ms to 424 ± 19 ms (*p* < 0.01). The mean increase in the QTc interval was greater after CQ than AQ-13: 28 ms (95% CI, 18 to 38 ms) versus 10 ms (95% CI, 2 to 17 ms). Figure 4 demonstrates the time course of the effects of the study drugs on the QTc interval, which then decreased gradually after day 2 as the AQ-13 and CQ blood levels fell. Despite prolongation of the QTc interval by both CQ and AQ-13, there were no cardiac AEs (Table 6).

When the data were analyzed by gender*,* the mean QTc prolongation tended to be greater with CQ than AQ-13 in both males and females (males: 16 ms for AQ-13, 95% CI 9 to 23 ms; 31 ms for CQ, 95% CI 16 to 46 ms; females: 12 ms for AQ-13, 95% CI 4 to 20 ms; 28 ms for CQ, 95% CI 17 to 39 ms). However, the small number of volunteers in each category did not permit statistical comparisons between males and females within drug groups or between drugs. As with the 600/700 mg dose, two volunteers who received the 1,500/1,750 mg dose developed QTc intervals > 450 ms 4 h after dosing on day 2 (453 ms for both). Both were female, both had received CQ; neither had any cardiac AEs.

Analysis of QTc interval changes at the individual level showed that the maximal prolongations of the QTc interval from baseline at the 600/700 mg dose were 54 ms for CQ and 42 ms for AQ-13. At the 1,500/1,750 mg dose, the maximal prolongation from baseline for CQ was 63 ms versus 35 ms for AQ-13. All four volunteers were female, and none experienced any cardiac AEs. QTc intervals returned to baseline in all participants by the time of the 2 wk follow-up.

## DISCUSSION

### Study Design and Interpretation

#### RCTs.

Because these studies were conducted as RCTs (Figure 1), they are different from Phase I clinical trials without controls. The rationale for this study design was twofold. First, the safety of CQ is sufficiently established that CQ is a standard against which other drugs are compared. Second, current expectations of the FDA and the public are markedly different today from the 1940s when CQ was first approved for clinical use [36,37]. Thus, there was a need to re-examine the safety and pharmacokinetics of CQ using strategies such as Holter monitoring that were not available 60 years ago.

As demonstrated in Table 1, the randomization strategy was effective: volunteers randomized to AQ-13 and CQ had similar age and sex distributions, similar weights and BMIs, and similar baseline QTc intervals. In terms of the upcoming Phase II studies of AQ-13 in Mali (West Africa), it is helpful that 37% (46/126) of the participants in Phase I were African or African American. The participation of these Africans and African Americans makes it less likely that the Phase II studies in Africa will identify new frequent AEs with AQ-13.

### Adverse Events

#### AEs during the GCRC inpatient stay.

Headache, lightheadedness, and GI tract AEs were reported most frequently; they occurred at similar frequencies with AQ-13 and CQ, and are known side effects of CQ [3,4,30–34]. Although AQ-13 may produce GI side effects more frequently (nausea, diarrhea), the number of volunteers studied does not permit one to conclude that AQ-13 has more GI toxicity than CQ. Other less common AEs, such as fatigue, blurred vision, ringing in ears, and rash were mild, transient, and had similar frequencies in both groups.

#### AEs identified during the follow-up visits.

At the 2 and 4 wk follow-up visits, there was no evidence for cardiac, ocular, hepatic, hematologic, renal, dermatologic, or other end-organ AEs. Although AEs involving these and other organs have been reported with AQs previously [31–34], they have typically been reported in persons treated for prolonged periods of time (5–10 y or more) at doses of 200–400 mg base or higher per day [34,38]. The absence of clinically detectable AEs and the normal laboratory tests in 119 volunteers at the 2 and 4 wk follow-up are consistent with previous reports on the safety of short-term CQ treatment [3,4,30–33]. The abnormal liver function test results (ALT, AST, and alkaline phosphatase) in one volunteer at the 300 mg dose may be related to AQ-13. However, all the tests were normal one week later and no similar hepatic AEs were observed in any volunteer with higher doses of AQ-13. The AEs observed are consistent with the hypothesis that AQ-13 is as safe as CQ in humans.

### Pharmacokinetics

Results obtained after the 600/700 and 1,500/1,750 mg oral doses of CQ and AQ-13 are consistent with previous studies; they demonstrated rapid oral absorption, a multiexponential decline in blood concentrations after the C_max_, a long terminal elimination t_1/2_, and a large Vd/F [39–41]. The estimated CQ clearance is also in agreement with previous reports [29,42]. However, accurate assessment of the terminal elimination t_1/2_ and the Vd/F is difficult because of tissue sequestration with CQ [29,40,42–44] and AQ-13. For example, the 14–24 d estimate of the terminal elimination t_1/2_ for CQ agrees with some reports [40,41], but is shorter than in others [29,42].

With the 700 mg dose, clearance of AQ-13 was less than with 600 mg (medians, 11.8 l/h versus 14.7 l/h; *p* = 0.01). One potential explanation is that participants who received the 700 mg dose were heavier than participants who received 600 mg (mean weights ± standard deviation of 83.3 ± 17.2 versus 72.0 ± 14.1 kg; *p* < 0.01). As a result, AQ-13 may have distributed more extensively in participants who received 700 mg because of extra body fat, which made the drug less available for elimination, and thus may have affected its clearance [43].

After 1,500 mg CQ, MDCQ was eliminated more slowly than CQ (MDCQ: terminal t_1/2_ of 21.8 d, MRT of 29.0 d; CQ: 13.2 and 16.0 d). In contrast, the terminal t_1/2_ and MRT of AQ-72 were similar to those of AQ-13 (Tables 4 and 5). The longer t_1/2_ and MRT of MDCQ (in comparison to CQ) are consistent with its lower renal clearance (3.8 l/h versus 6.0 l/h; *p* = 0.03), and with the findings of other investigators [41,42,45]. As with MDCQ and CQ, the renal clearance of AQ-72 was less than that of its parent compound, AQ-13 (2.0 l/h versus 3.3 l/h; *p* < 0.01). However, the similar t_1/2_ values and MRTs of AQ-72 and AQ-13 are inconsistent with the lower Cl_r_ of AQ-72; these findings suggest that another pathway, such as metabolism of AQ-72 to AQ-73 by the CYP450 system, may account for this difference. The greater urinary excretion of AQ-13 and CQ than their more water-soluble metabolites (Tables 4 and 5) [26] is consistent with the active transport of CQ, and possibly AQ-13, by organic cation transporters such as organic cation transporter-like 2 (ORCTL2) [46]. The paradoxical observation that AQ-72 has both a shorter MRT in the blood and a lower Cl_r_ than MDCQ (Table 5) may be explained by a greater role for CYP450 metabolism (*N-*dealkylation) with AQ-13 than CQ [47]; this hypothesis is also consistent with the observation that the urinary ratio for AQ-73/AQ-13 was twice the urinary ratio for BDCQ/CQ, consistent with greater conversion of AQ-72 to AQ-73 than of MDCQ to BDCQ.

### Effects of AQ-13 and CQ on the QTc Interval

Previous animal [48,49] and human studies [28,50,51] have shown that CQ prolongs the QT interval. The results reported here confirm those observations, and establish the dose (blood-level)-related nature of QTc prolongation by CQ. At the 600 mg dose, CQ prolonged the mean QTc interval by 15 ms (Table 6). The same effect (16 ms QTc prolongation) was seen 4 h after the first 600 mg CQ dose (on day 1) with the 1,500 mg therapeutic dose of CQ (Figure 4). The QTc interval increased by an additional 12 ms after the second 600 mg CQ dose on day 2 (mean increase of 27 ms relative to baseline), and then decreased gradually as CQ blood levels fell after the third (300 mg) dose on day 3, and thereafter, thus demonstrating a dose (blood level)–response relationship between the CQ blood level and QTc prolongation. These results are consistent with a previous study that suggested a dose-dependent effect of CQ on the QT interval after oral administration [28].

Although a similar pattern was observed with AQ-13, the effects of AQ-13 on the QTc interval were less than those of CQ. For example, the first 700 mg dose at the 1,750 mg level prolonged the mean QTc interval by 4 ms, and the second by an additional 6 ms. The QTc interval then decreased gradually thereafter as the AQ-13 blood levels fell (Figure 4; Table 6).

When the effects of AQ-13 and CQ were analyzed by gender, QTc prolongation was significant only for females after the 600 and 700 mg doses. In contrast, significant QTc prolongation was observed in both males and females after the 1,500/1,750 mg dose (Table 6). This discrepancy could be due to the known increased vulnerability of women to drug-induced QTc interval prolongation [52,53], which caused this effect to appear in them at doses lower than in men; alternatively, this could be a chance finding because of the small sample sizes involved. These results establish that AQ-13, like CQ, prolongs the QTc interval in humans and that CQ produces greater QTc prolongation than AQ-13. However, the significance of these observations is unclear because no arrhythmias or other cardiac AEs were observed in any participants.

### Generalizability

The results reported here suggest that the AEs of AQ-13 may be no different from those of CQ, that higher doses of AQ-13 than CQ may be necessary to produce similar blood levels and AUCs, and that AQ-13 may produce less QT prolongation than CQ in humans. However, given the small numbers and nonrepresentative selection of study participants, the extent to which these results are generalizable is unclear.

### Overall Evidence

The results reported here are consistent with the hypotheses underlying the objectives of these studies. First, the similar AEs observed with AQ-13 and CQ are consistent with the hypothesis that AQs with structures similar to CQ should be similarly safe in humans. Second, they demonstrate that AQ-13, an AQ analogous to CQ, has similar linear pharmacokinetics in human volunteers, despite the fact that it requires a larger dose to achieve equivalent drug exposure because of a more rapid clearance. These results are also consistent with the preclinical studies, which suggested that the AEs of AQ-13 and CQ would be similar and that a dose adjustment would be necessary for AQ-13 because of its more rapid clearance [17,18]. Because this Phase I study has demonstrated the safety of AQ-13 doses up to 1,750 mg, the next logical study (after examining the effects of a fatty meal on the absorption of AQ-13) is a dose-finding efficacy (Phase 2) study in humans with uncomplicated P. falciparum malaria.

## SUPPORTING INFORMATION

CONSORT Checklist Click here for additional data file. (65 KB DOC)

Trial Protocol Click here for additional data file. (32 KB DOC)

Alternative Language Abstract S1 Click here for additional data file. Translation of the Abstract into Arabic by Fawaz Mzayek
(27 KB DOC)

Alternative Language Abstract S2 Click here for additional data file. Translation of the Abstract into Chinese by Haiyan Deng
(88 KB PDF)

Alternative Language Abstract S3 Click here for additional data file. Translation of the Abstract into French by Ousmane Koita and Fawaz Mzayek
(28 KB DOC)

Alternative Language Abstract S4 Click here for additional data file. Translation of the Abstract into German by Christiane Hadi
(32 KB DOC)

Alternative Language Abstract S5 Click here for additional data file. Translation of the Abstract into Japanese by Kaori and Hitosi Tanaka
(85 KB PDF)

Alternative Language Abstract S6 Click here for additional data file. Translation of the Abstract into Russian by Maya Begalieva
(31 KB DOC)

Alternative Language Abstract S7 Click here for additional data file. Translation of the Abstract into Spanish by Juan Lertora
(22 KB DOC)

## Abbreviations

AE: adverse event
ALT: alanine aminotransferase
AQ: aminoquinoline
AST: aspartate aminotransferase
AUC: area under the curve
AUCw1: partial area under the curve for week 1
AUCτ: total area under the curve
BMI: body mass index
CI: confidence interval
Cl/F: oral clearance
Clr: renal clearance
C_max_: maximal drug concentration in the blood
CQ: chloroquine
ECG: electrocardiogram
GCRC: Tulane–Louisiana State University–Charity Hospital General Clinical Research Center
GI: gastrointestinal
GMP: Good Manufacturing Practice
HPLC: high-performance liquid chromatography
LSU: Louisiana State University
MRT: mean residence time
PK: pharmacokinetic(s)
QTc: QT interval adjusted for a heart rate different from 60 beats per minute
RCT: randomized controlled trial
t1/2: half-life
T_max_: time from oral administration to C_max_
Vd/F: apparent volume of distribution
