# Supplementary material for: Randomized Dose-Ranging Controlled Trial of AQ-13, a Candidate Antimalarial, and Chloroquine in Healthy Volunteers
Source: PLoS Clin Trials. 2007 Jan 5;2(1):e6. doi: 10.1371/journal.pctr.0020006 (PMC1764434; doi:10.1371/journal.pctr.0020006)
Supplement: Trial Protocol [file pctr.0020006.sd002.doc]

**Phase 1 Studies of AQ-13, a Candidate Aminoquinoline Antimalarial, in Comparison With Chloroquine**

**Study Protocol**

**Purpose:**

The purpose of this protocol is to perform Phase 1 (safety/toxicity and pharmacokinetic) Studies of an investigational aminoquinoline antimalarial (AQ-13) in human subjects. The compound to be studied (AQ-13) is being examined because it is active *in vitro* against *Plasmodium falciparum* malaria parasites resistant to chloroquine (CQ) and other antimalarials (multi-resistant *P. falciparum*), and because its safety was similar to that of CQ in preclinical studies performed by SRI International (IND 55,670). AQ-13 was also selected for study because it is active in vivo in two monkey models of human malaria: 1] *P. cynomolgi* in the rhesus monkey (*Macaca mulatta*), a model of human infection with *P. vivax*, and 2] CQ-resistant *P. falciparum* in the squirrel monkey, a model of human infection with CQ-resistant *P. falciparum*.

**Study Population:**

Healthy young men and women 21-45 years of age who are taking no chronic medications with the exception of birth control pills will be invited to participate in this Phase 1 Study at the Tulane/LSU/Charity Hospital General Clinical Research Center (GCRC) in New Orleans. Exclusion criteria include pregnancy, breast feeding, abnormal liver or kidney function tests, anemia (Hb < 12 gm per dL), chronic medications other than birth control pills, and an abnormal baseline ECG or Holter recording. Because the purpose of this testing is to determine whether AQ-13 is likely to have significant toxicity in Africans (Malians), ≥ 25% of the volunteers studied in New Orleans will be African-Americans.

**Randomization:**

Drug allocation codes will be generated by the study biostatistician in blocks of size 4 and 6, using computer software, and will be sealed in numbered, opaque envelopes. Block sizes will be determined at random so that they will not be known to the study personnel. The envelopes containing the randomization codes will be hand-delivered to the study pharmacist and kept in the Research Pharmacy, which is outside the GCRC.

**Blinding:**

The study participants, investigators and staff will be blinded to the drug administered (AQ-13 or CQ) to subjects at each stage of the study. The interim reports to the DSMB after completion of each dose level will be presented without breaking the code, unless deemed necessary by the DSMB. The envelope containing the drug allocation code will be opened by the study pharmacist and the appropriate drug will be dispensed to the GCRC on the morning of its administration. With the exception of the 600 mg CQ tablets (Aralen™) tablets, the two drugs (AQ-13 and CQ) will be administered in identical capsular form and number.

**Informed Consent:**

Informed consent will be obtained from each participant before screening. Based on the IRB guidelines, the informed consent form will be reviewed and updated at yearly intervals, and whenever new pertinent information on the study drugs or their side effects becomes available.

**Baseline Screening:**

To determine their eligibility, each volunteer will have a complete physical exam, including an eye examination (visual acuity, visual fields, indirect ophthalmoscopy), panels of standard chemical tests (BUN, Creatinine, AST, ALT, LDH, Alkaline Phosphatase, Glucose, Bilirubin, Creatine Kinase) and hematologic tests (Hematocrit, Hemoglobin, White Cell Count and Platelet Count), and a cardiac examination (physical exam, baseline ECG and 24-hour Holter recording) for arrhythmias and other evidence of cardiac disease.

**In-patient Studies at the Tulane-LSU-Charity Hospital GCRC:**

Volunteers will be hospitalized the night before drug administration at the GCRC, prior to randomization to receive either AQ-13 or CQ capsules orally (*po*) the next morning. Doses will begin at 10 mg base with 8 volunteers per drug x dose group, and will escalate in subsequent groups to 100, 300 and 600 mg base (8 subjects per drug x dose group for the 10, 100 and 300 mg doses [subtotal of 48 subjects]; 12 subjects per drug x dose group at the 600 mg dose in order to compare the pharmacokinetics of CQ and AQ-13 before proceeding to the equivalent therapeutic dose). At the request of FDA, a third group will be added at the 600 mg dose to determine whether the blood levels obtained with CQ capsules are equivalent to the blood levels obtained with commercially available FDA-approved Sanofi-Winthrop CQ tablets (Aralen™, i.e., 12 subjects per drug x dose group at 600 mg x 3 drug groups [AQ-13 capsules, CQ capsules and Aralen™ CQ tablets] = subtotal of 36 subjects]).

To compare the absorption and metabolism of AQ-13 with the absorption and metabolism of CQ, blood samples will be obtained for AQ-13, CQ and metabolite blood levels immediately before and 1, 2, 4, 6, 8, 12, 18, 24, 36, 48, 60, 72, 84, 96 and 120 hours after the 600 and the 1500 mg doses using a heparin lock. At the 1500 mg dose, three 24-hour urine collections will be obtained on days 1-3 after beginning dosing on day 1 to compare the urinary excretion of AQ-13, CQ and their metabolites. Volunteers receiving 600 and 1500 mg doses of AQ-13 or CQ will return for blood samples twice weekly during the ensuing 4 weeks to define the terminal half-lives of AQ-13, CQ and their metabolites. After measurement of AQ-13, CQ and metabolite levels following the 600 mg dose, the doses given in the second set of studies (equivalent therapeutic course – i.e., 1500 mg CQ) will be adjusted (Dose Adjustment, see below) to obtain blood levels of AQ-13 similar to those obtained with CQ. In these studies, volunteers will receive the equivalent of 600 mg CQ base on the mornings of days 1 and 2, and an additional 300 mg CQ base equivalent on day 3.

Previous experience with CQ (a structurally similar aminoquinoline) suggests that massive overdoses of AQ-13 may produce arrhythmias, although there has been no previous human experience with AQ-13. Although the arrhythmiagenic effects of CQ have been reported only with massive ingestions or rapid intravenous infusions, these Phase 1 Studies will provide an excellent opportunity to test for this potential toxicity. Therefore, we will use Holter monitoring during the Phase 1 Studies in New Orleans to ensure that there is no evidence of arrhythmias with AQ-13. Conventional electrocardiograms will be used to test for the T wave flattening and QTc prolongation, typically seen in persons receiving therapeutic courses of CQ. Continuous Holter monitoring will be performed to evaluate the effects of AQ-13 and CQ on the QT interval after the 1500 mg dose.

**Participants’ Out-patient Follow-ups:**

After discharge from their in-patient stay at the GCRC (2½ days for the 10, 100, 300 and 600 mg doses; 3½ days for the 1500 mg dose), participants will be asked to return to the GCRC twice weekly for a total of 4 weeks after discharge in order to obtain blood for drug and metabolite blood levels, and for the evaluation of adverse events (AEs). An ECG and chemistry and hematology lab tests will be repeated at the 2 week and 4 week follow-up visits.

**Dose Adjustment for AQ-13:**

The data obtained by SRI International during GLP preclinical toxicologic and pharmacokinetic studies indicate that the oral bioavailability of AQ-13 is less than that of CQ in rats and monkeys. Therefore, it is possible (perhaps likely) that the oral bioavailability of AQ-13 in humans will be less than that of CQ, and thus that it may be necessary to increase the oral dose of AQ-13 in order to provide molar blood levels of AQ-13 similar to those produced by the established oral doses of CQ. To estimate the amount of AQ-13 necessary to obtain similar oral bioavailability, we will compare blood levels and areas under the curve (AUCs) for AQ-13 and CQ at the 600 mg dose. Based on these results, we will estimate the dose adjustment (increment or decrement) necessary for AQ-13 and test that adjustment in 12 additional volunteers. After the dose of AQ-13 necessary to produce equimolar blood levels and AUCs has been established, we will compare that adjusted dose (equivalent therapeutic dose) of AQ-13 to 1500 mg CQ base.
