# Supplementary material for: Randomized Dose-Ranging Controlled Trial of AQ-13, a Candidate Antimalarial, and Chloroquine in Healthy Volunteers
Source: PLoS Clin Trials. 2007 Jan 5;2(1):e6. doi: 10.1371/journal.pctr.0020006 (PMC1764434; doi:10.1371/journal.pctr.0020006)
Supplement: Alternative Language Abstract S1 [file pctr.0020006.sd003.doc]

**ملخّص البحث:**

أهداف الدراسة: 1) تحديد الحركيات الدوائية (pharmacokinetics) والسلامة السريرية لـ (AQ-13) وهو دواء تجريبي جديد من مجموعة الأمينوكينولينات (aminoquinolines) مضاد للسلالات المقاومة من طفيلي الملاريا (Plasmodium) والتي تسبّب الملاريا المعنّدة على العلاج. 2) مقارنة الحركيات الدوائية والآثار الجانبية، بما فيها التأثير على زمن QT للقلب، بين الـ (AQ-13) ودواء الكلوروكين (= CQ chloroquine).

منهج الدراسة: أجريت الدراسة بطريقة الاختبار السريري العشوائي مضاعف التعمية. تمّ توزيع المتطوعين بشكل عشوائي إلى مجموعتين، أعطيَت إحداها (AQ-13) وأعطيَت الأخرى جرعة مكافئة من الـ (CQ) صباحاً على معدة خالية. كررت التجربة على عدة مراحل بدءاً من جرعة 10 ملغ، 100 ملغ، 300 ملغ، 600 ملغ وحتى 1500 ملغ. أجريَت الدراسة في "المركز المشترك للتجارب السريرية لجامعتي Tulane و LSU" في الولايات المتحدة الأمريكية.

عينة الدراسة: 126 متطوعاً صحيحي البنية بين 21 و 45 سنة من العمر.

المتغيرات التابعة: تتضمّن التأثيرات الجانبية المخبرية والسريرية للدوائين، المؤشرات الحركية الدوائية، تأثير الدواء على دورة الاستقطاب البطيني للقلب مقاسة بالتغير الحاصل في زمن QT على مخطط القلب الكهربائي.

النتائج: لم تظهر أية تأثيرات سمّية دموية أو كبدية أو كلوية أو عينية أو في أعضاء أخرى في أي من المجموعتين. أكثر الأعراض الجانبية ملاحظةً كانت على الترتيب: صداع (17 متطوعاً من 63 في مجموعة الـ AQ-13 و 10 متطوعين من 63 في مجموعة الـ CQ؛ *p* = 0.2)، دوار (63/11 للـ AQ-13 و 63/8 للـ CQ؛ *p* = 0.6)، أعراض متعلقة بالجهاز الهضمي: غثيان، فقد شهية، إقياء، إسهال، ألم بطني ( 63/14 للـ AQ-13 و 63/13 للـ CQ). كلا الدوائين أظهرا حركيات دوائية خطّية وحجم انتشار ظاهري (Vd/F) متماثل. ولكن AQ-13 أبدى سرعة انطراح ظاهري (Cl/F) أكبر من CQ (14-14.7 ل/سا لـ AQ-13 مقابل 9.5-11.3 ل/سا لـ CQ؛ *p* ≤ 0.03). زمن QT على المخطط الكهربائي للقلب أبدى تطاولاً أكبر في المتوسط في المجموعة التي تناولت ال CQ منه في المجموعة التي تناولت ال AQ-13 (28 ميليثانية مع 95% مدى ثقة = 18، 38 ميليثانية، مقابل 10 ميليثانية مع 95% مدى ثقة = 2، 17 ميليثانية؛ *p* = 0.01).

الاستنتاج: أظهرت هذه الدراسات فروقاً طفيفة في السميّة الدوائية بين الدواء التجريبي AQ-13 و الكلوروكين، كما بيّنت أن الدوائين يمتلكان حركيات دوائية متماثلة.
