# Supplementary material for: Randomized Dose-Ranging Controlled Trial of AQ-13, a Candidate Antimalarial, and Chloroquine in Healthy Volunteers
Source: PLoS Clin Trials. 2007 Jan 5;2(1):e6. doi: 10.1371/journal.pctr.0020006 (PMC1764434; doi:10.1371/journal.pctr.0020006)
Supplement: Alternative Language Abstract S2 [file pctr.0020006.sd004.pdf]

PLoS 临床试验摘要

摘要

试验目的:1).检测研究氨基喹啉(AQ-13)活性抑制虐疾寄生虫的药物动力学和安全性,包括它在 QRS 波到 T 波的 间距的影响. 2) 检测应用在人体上, 氨基喹啉(AQ-13)是否和氯喹(CQ)有相似的药物动力学和安全性.

试验设计:这个设计做为第一阶段的研究是双重盲目的,在健康的志愿者身上使用随机法对照试验去对比氨基喹啉(AQ-13) 和氯喹(CQ).预先的药剂量设计完成之后,每一步的试验都是随记选择完成的.

试验设置:住院病人和非住院病人的观察研究都是在新奥尔良杜兰------路易斯安纳州------慈爱医院普通临床研究中心完成的.

试验参予者:共有 126 名健康的年龄在 21-45 岁的成人参予试验.

试验的调整: 试验设计为 10, 100, 300, 600 和 1500 毫克口服剂量的氯喹盐对照相等剂量的氨基喹啉(AQ-13).

试验结果的测量:包括临床和实验室中的不利的事件, 药物动力学参数和对心脏的影响(QT 的延长值).

试验结果:使用氨基喹啉(AQ-13)或氯喹(CQ),在任何剂量的药物测试中,都没有发现关于血液方面的,肝脏的,肾脏的, 视觉的或其它器官的毒性.头痛, 头昏眼花/晕眩和肠胃道的症状(恶心,没食欲,呕吐,痢疾,腹痛)是最普通的副反应.虽然这些症状在使用氨基喹啉(AQ-13)时是比较频繁发生的,但是对于使用氨

基喹啉(AQ-13)和氯喹(CQ), 产生这些症状的志愿者人数是相似的.(氨基喹啉(AQ-13)和氯喹(CQ)的结果分别为: 头痛:17/63 和 10/63,p=0.2; 头昏眼花/晕眩:11/63 和 8/63, p=0.6; 肠胃道症状:14/63 和 13/63, p=0.9). 氨基喹啉(AQ-13)和氯喹(CQ)都展示了线性的药物动力学和相似的表面分布体积(Vd/F).然而,相对于氯喹(CQ), 氨基喹啉(AQ-13)可以比较快速地从人体中被清除(CL/F 中值分别为 14-14.7 相对 9.5-11.3, p≤0.03). 相对于氨基喹啉(AQ-13), 氯喹(CQ)的使用产生比较大的 QT 延长值.(氯喹(CQ):平均增长 28 毫秒;95%CI=18,38 毫秒的产生是从 396 毫秒到 424 毫秒, p=0.01. 氨基喹啉(AQ-13): 平均增长 10 毫秒;95%CI=2,17 毫秒的产生是从 397 毫秒到 407 毫秒, p=0.01).无论使用氨基喹啉(AQ-13)或氯喹(CQ),都没有发现心律不齐或其它心脏方面的副反应,

试验总结:这些研究表明氨基喹啉(AQ-13)和氯喹(CQ)之间在毒性方面有最小的差异和相似的药物动力学.

试验登记: 临床试验.管理; 试验登记号:NCT00323375; 试验网址:http://www.clinicaltrials.gov/ct/show/NCT00323375?order-1.
