# Supplementary material for: Randomized Dose-Ranging Controlled Trial of AQ-13, a Candidate Antimalarial, and Chloroquine in Healthy Volunteers
Source: PLoS Clin Trials. 2007 Jan 5;2(1):e6. doi: 10.1371/journal.pctr.0020006 (PMC1764434; doi:10.1371/journal.pctr.0020006)
Supplement: Alternative Language Abstract S3 [file pctr.0020006.sd005.doc]

**Résumé**

**Objectifs:** déterminer: 1] la pharmacocinétique et la sécurité d'une nouvelle aminoquinoléine (AQ-13) active contre les parasites résistants du paludisme, en incluant ses effets sur la prolongation de l’intervalle QTc, et 2] si l’AQ-13 aurait un profil pharmacocinétique et sécuritaire similaire à la chloroquine (CQ) chez les humains**.**

**Protocole:** Les études ont été faites comme une étude de Phase 1 : un essai clinique randomisé et contrôlé (à double aveugle) pour comparer AQ-13 et CQ chez des volontaires en bonne santé. Les randomisations ont été respectées à chaque étape après la fin de la précédente dose.

**Location:** Les études ont été exécutées au General Clinical Research Center (GCRC) de Tulane-LSU-Charity Hospital à la Nouvelle Orléans.

**Participants**: 126 adultes en bonne santé entre 21 et 45 ans.

**Interventions:** les doses étaient de 10, 100, 300, 600 et 1500 mg de CQ en comparaison avec des doses équivalentes d’AQ-13.

**Mesures obtenues** incluaient les effets adverses cliniques et biologiques, les paramètres pharmacocinétiques et les effets cardiaques (prolongation d'intervalle de QTc).

**Résultats:** Il n’y avait pas de toxicité par l’AQ-13 ou le CQ au niveau hematologique, hépatique, rénal, oculaire et autres organes à aucune des doses testées. Maux de tête, étourdissements/vertiges, et symptômes gastro-intestinaux (nausées, perte de l’appétit, vomissements, diarrhée, douleur abdominale) étaient effets secondaires les plus communs. Bien que les symptômes étaient plus fréquents avec AQ-13, le nombre de volontaires qui ont eu des symptômes avec AQ-13 et CQ était similaire (Maux de tête: 17/63 et 10/63, *p* = 0.2; étourdissement/vertige: 11/63 et 8/63, *p* = 0.6; symptômes gastro-intestinaux 14/63 et 13/63, *p* = 0,9, pour AQ-13 et CQ, respectivement). **AQ-13 et CQ ont montré une pharmacocinétique linéaire, et avaient des volumes apparents de distribution (Vd/F) similaires. Cependant, AQ-13 a été éliminé plus rapidement que CQ (Cl/F médiane 14-14.7 vs 9.5-11.3 L/hr, *p* < 0.03).** La prolongation de QTc était plus importante avec CQ qu’avec AQ-13 (augmentation de 28 msec; 95% CI = 18, 38 msec pour CQ, de 396 à 424 msec, vs. augmentation de 10 msec; 95% CI = 2, 17 msec pour AQ-13, de 397 à 407 msec, *p* = 0.01). Il n’y a pas eu d’arythmie ou d’autre événements adverses cardiaques, ni avec AQ-13 ni avec CQ.

**Conclusion:** Ces études révèlent des différences de toxicité minimales entre AQ-13 et CQ, et une similaire pharmacocinétique.
