# Supplementary material for: Randomized Dose-Ranging Controlled Trial of AQ-13, a Candidate Antimalarial, and Chloroquine in Healthy Volunteers
Source: PLoS Clin Trials. 2007 Jan 5;2(1):e6. doi: 10.1371/journal.pctr.0020006 (PMC1764434; doi:10.1371/journal.pctr.0020006)
Supplement: Alternative Language Abstract S4 [file pctr.0020006.sd006.doc]

***ABSTRAKT FÜR PLoS CLINICAL TRIALS.***

**ABSTRAKT**

**Fragestellung:** Bestimmung von 1] der Pharmakokinetik und Sicherheit eines neuen Aminoquinolines welches Aktivität gegen resistente Malaria Parasiten besitzt (AQ-13), einschliesslich des Effekts auf den QTc Interval und 2] ob das pharmakokinetische - und Sicherheitsprofil von AQ-13 dem von Chloroquin (CQ) im Menschen aehnlich ist.

**Studienentwurf:** Diese Studie wurde als doppel-blind, randomisierte, kontrollierte Phase 1 Studie konzipiert, um AQ-13 und CQ in gesunden Voluntären zu vergleichen. Randomisierung erfolgte nach jedem Studienabschnitt nach Beendigung der Untersuchungen der vorhergehenden Dosis.

**Studienort:** Stationäre und ambulante Untersuchungen wurden im Tulane-LSU-Charity Hospital General Clinical Research Center in New Orleans durchgeführt.

**Teinehmer** waren 126 gesunde Erwachsene im Alter von 21 bis 45 Jahren.

**Interventionen** waren 10, 100, 300, 600 und 1500 mg orale Dosen von CQ Basis im Vergleich zu äquivalenten Dosen von AQ-13.

**Messung der Ergebnisse** umfassten klinische unerwünschte Ereignisse, Laborwerte, pharmakokinetische Parameter und Auswirkungen auf das Herz (QT Verlängerung).

**Ergebnisse:** Es wurden keine toxischen Effekte auf das hämatologische System, die Leber, die Nieren, die Augen oder andere Organe mit den getesteten Dosen von AQ-13 und CQ nachgewiesen. Kopfschmerzen, Schwindel und gastrointestinale Symptome (Übelkeit, Appetitsverlust, Erbrechen, Durchfall, Bauchschmerzen) waren die häufigsten unerwünschten Ereignisse. Obwohl diese Symptome mit AQ-13 etwas häufiger waren, unterschied sich die Anzahl der Voluntäre mit diesen Symptomen in der AQ-13 Gruppe und der CQ Gruppe nicht (Kopfschmerzen: 17/63 und 10/63, *p* = 0.2; Schwindel: 11/63 and 8/63, *p* = 0.6; Gastrointestinale Symptome 14/63 and 13/63, *p* = 0.9, für AQ-13 and CQ). AQ-13 und CQ zeigten lineare Pharmakokinetik und ähnliche Verteilungsvolumina. AQ-13 wurde schneller als CQ ausgeschieden **(CL/F Median 14-14.7 vs 9.5-11.3, p < 0.03).** QTc Verlängerung war grösser mit CQ als mit AQ-13 (durchschnittlicher Anstieg von 28 msec; 95% CI = 18, 38 msec für CQ von 396 nach 424 msec vs. durchschnittlicher Anstieg von 10 msec; 95% CI = 2, 17 msec für AQ-13 von 397 nach 407 msec, *p* = 0.01). Es traten keine Arrhythmien oder andere kardielle unerwünschte Ereignisse auf mit AQ-13 oder CQ.

**Schlussfolgerungen:** Diese Studie hat minimale Unterschiede in der Toxizität und ähnliche Pharmakokinetik von AQ-13 und CQ nachgewiesen.

**Studienregistrierung:** ClinicalTrials.gov; Registriernummer: NCT00323375; URL für Studie: <http://www.clinicaltrials.gov/ct/show/NCT00323375?order-1>.
