# Supplementary material for: Randomized Dose-Ranging Controlled Trial of AQ-13, a Candidate Antimalarial, and Chloroquine in Healthy Volunteers
Source: PLoS Clin Trials. 2007 Jan 5;2(1):e6. doi: 10.1371/journal.pctr.0020006 (PMC1764434; doi:10.1371/journal.pctr.0020006)
Supplement: Alternative Language Abstract S5 [file pctr.0020006.sd007.pdf]

ABSTRACT FOR PLoS CLINICAL TRIALS.

要旨

目的：1] 研究中の抗マラリア原虫 aminoquinoline(AQ-13)の薬物動態及びその安全性と効果時間 OTc、2]AQ-13 が人体に於いて chloroquine（CQ）と同等の薬物動態特性と安全特性を持つかどうかを確定する。

企画：この AQ-13 と CQ の比較研究はフェーズ 1 として、健康な成人ボランティア達の二重盲検法、無作意抽出試験(Randomized Controlled Trials=RCTs)を企画した。無作意抽出は各段階に於いて前段の投与後に実施した。

環境設定：入院患者及び外来患者に対する研究はニューオリンズの Tulane-LSU-Charity Hospital General Clinical Research Center に於いて実施した。

参加者は 21～45 歳の健康な成人 126 名。

推移比較は CQ と AQ-13 について 10,100,300,600,1500mg のそれぞれの経口投与で行った。

測定結果には臨床及び実験での副作用、薬物動態範囲及び心臓に対する影響（QT prolongation）を含めた。

結果：AQ-13 又はCQの投与による血液系、肝機能、腎機能、視覚系、その他臓器に対する毒性は認められなかった。頭痛、めまい及び消化器系関連症状（吐き気、食欲減退、嘔吐、下痢、腹痛）が最も共通した副作用であった。これらの症状はAQ-13 の方が依り頻度が高かったが、CQに於いても類似であった。（AQ-13/CQ対比： 頭痛：17/63 及び 10/63、p=0.2、めまい：11/63 及び 8/63、p=0.6、GI症状：14/63 及び 13/63、p=0.9） AQ-13 とCQの両方に於いて直線的な薬物動態および同等な体内分布が顕わとなった。

しかしながら、AQ-13 はCQより速く排泄された。（CL/F中点：14-14.7 対 9.5-11.3、p ≤ 0.03)。

持続性 QTc は CQ の方が AQ-13 より大であった。(CQ： 396 から 424 msec に於いて 95% CI=18、38 msec で平均 28 msec 増加。一方 AQ-13：397 から 407msec に於いて 95% CI = 2、 17 msec、で 10 msec 平均増加、*p* = 0.01)

不静脈やその他心臓疾患は AQ-13、CQ 共に見られなかった。

結論：本研究によって AQ-13 と CQ 間の毒性度は微差であること並びに類似の薬物動態特性を持つことが明確となった。

試験登録：臨床試験登録番号：NCT00323375
URL：http://www.clinicaltrials.gov/ct/show/NCT00323375?order-1.
