# Supplementary material for: Randomized Dose-Ranging Controlled Trial of AQ-13, a Candidate Antimalarial, and Chloroquine in Healthy Volunteers
Source: PLoS Clin Trials. 2007 Jan 5;2(1):e6. doi: 10.1371/journal.pctr.0020006 (PMC1764434; doi:10.1371/journal.pctr.0020006)
Supplement: Alternative Language Abstract S6 [file pctr.0020006.sd008.doc]

**Тезиз: Первая фаза исследования потенциального антималярийного препарата, АК-13**

Цель: Определить 1) фармакокинетику и безопасность применения аминоквинолина-13 (АК-13) против резистентных паразитов, вызывающих малярию, влкючая его еффект на интервал QT, и 2) является ли фармакокинетика АК-13 похожей на фармакокинетику хлороквина в человеческом организме.

План: Это исследование является 1-й фазой дважды-скрытого рандомизированного исследования, целью которого является сравнение действия АК-13 и хлороквина в здоровом организме. Рандомизация проводилась после введения каждой дозы.

Место исследования: Амбулаторные и стационарные исследования были проведены в Клиническом Исследовательском центре Университета Тюлейн, Университета Штата Луизиана и Благотворительной Больницы города Новый Орлеан.

Дозы приема были 10, 100, 300, 600 и 1500 мг. оральных доз как хлороквина, так и АК-13

Участники исследования: 126 взрослых участников в возрасте от 21 до 45 лет.

Измерение последствий включали в себя клинические и лабораторные неблагоприятные побочные действия, параметры фармакокинетики и кардиальные эффекты (продление интервала QT).

Результаты: Токсичность на печень, почки, глаза и органы кроветворения не наблюдалась при применении вышеуказанных доз АК-13 или хлороквина. Наиболее общие неблагоприятные побочные эффекты были головная боль, головокружение и желудочно-кишечные симптомы (тошнота, отсутствие аппетита, рвота, диарея, боли в животе). Хотя симптомы наблюдались чаще при применении АК-13, количество участников с симптомами было одинаковое в обеих группах (головная боль: 17/63 и 10/63, р= 0,2; головокружение: 11/63 и 8/63, р=0,6; желудочно-кишечные симптомы: 14/63 и 13/63, р=0,9, для АК-13 и хлороквина, соответсвенно). Оба АК-13 и хлороквин продемонстрировали линейную фармакокинетику и показали одинаковые объемы распределения (Vd/F). Однако АК-13 выводился из организма быстрее, чем хлороквин (среднее хлороквин/F 14-14,7 в сравнении 9,5-11,3, р.≤0,03). Продление интервала QT наблюдалось больше с применением хлороквина, нежели АК-13 (среднее увеличение на 28 мсек; 95% доверительный интервал = 18, 38 мсек для хлороквина от 396 до 424 мсек в сравнении со среднем увеличением на 10 мсек; 95% доверительный интервал = 2,17 мсек для АК-13 от 397 до 407 мсек, р=0,01). Аритмия или другие наблагоприятные действия не наблюдались при применении АК-13 и хлороквина.

Заключение: Исследования выявили одинаковую фармакокинетику и минимальные различия в токсичности между АК-13 и хлороквином.

Регистрация исследования: ClinicalTrials.gov; Регистрационный номер: NCT00323375; URL: <http://www.clinicaltrials.gov/ct/show/NCT00323375?order-1>.
