# Supplementary material for: Randomized Dose-Ranging Controlled Trial of AQ-13, a Candidate Antimalarial, and Chloroquine in Healthy Volunteers
Source: PLoS Clin Trials. 2007 Jan 5;2(1):e6. doi: 10.1371/journal.pctr.0020006 (PMC1764434; doi:10.1371/journal.pctr.0020006)
Supplement: Alternative Language Abstract S7 [file pctr.0020006.sd009.doc]

**AQ-13 versus Cloroquina: Estudio Controlado Randomizado**

**Objetivos**: 1. Establecer el perfil farmacocinético y la seguridad de una aminoquinolina experimental (AQ-13) activa contra malaria resistente, incluyendo sus efectos sobre el intervalo QTc. 2. Determinar si AQ-13 tiene un perfil farmacocinético y de seguridad similar a la cloroquina (CQ).

**Diseño**: Estudio Fase 1, doble ciego, controlado, randomizado comparando AQ-13 y CQ en voluntarios sanos.

**Métodos**: Para realizar estudios farmacocinéticos los pacientes se admitieron durante 24-48 hs en el Centro de Investigación Clínica común a las Universidades de Tulane, del Estado de Louisiana, y del Hospital de Caridad en Nueva Orleans. Posterior al alta los pacientes continuaron siendo evaluados en forma ambulatoria.

**Participantes**: 126 personas adultas sanas de 21-45 años de edad residentes de la ciudad de Nueva Orleans.

**Intervenciones**: Se compararon dosis de CQ base de 10, 100, 300, 600 y 1500 mg por vía oral con dosis equivalentes de AQ-13.

**Evaluaciones**: Efectos adversos (EAs) clínicos y de laboratorio, parámetros farmacocinéticos y efectos cardíacos (prolongación del intervalo QT) fueron evaluados a lo largo del estudio..

**Resultados**: No se observaron toxicidades hematológicas, hepáticas, renales, oculares o de otra índole con AQ-13 ni CQ en las dosis estudiadas. Cefalea, mareos y síntomas gastrointestinales (GI) (nausea, anorexia, vómitos, diarrea y dolor abdominal) fueron los EAs mas comunes. El número de voluntarios que reportaron efectos adversos con AQ-13 y CQ fue similar (cefalea: 17/63 y 10/63, p = 0.2; mareo: 11/63 y 8/63, p = 0.6; GI: 14/63 y 13/63, p = 0.9, para AQ-13 y CQ, respectivamente). Tanto AQ-13 como CQ exhibieron una farmacocinética linear y tuvieron volúmenes de distribución aparente (Vd/F) comparables. Sin embargo, el clearance de AQ-13 fue mas rápido que el de CQ (mediana de CL/F 14-14.7 versus 9.5-11.3, p < 0.03). La prolongación del QTc fue de mayor magnitud con CQ que con AQ-13 (aumento promedio de 28 mseg; 95% IC = 18-38 mseg para CQ de 396 a 424 mseg versus aumento promedio de 10 mseg; 95% IC = 2-17 mseg para AQ-13, de 397 a 407 mseg, p = 0.01). No se observaron arritmias ni otros EAs cardíacos con ninguna de las dos drogas.

**Conclusiones**: Este estudio revelo mínimas diferencias en toxicidad entre AQ-13 y CQ. Excepto por las diferencias en clearance, ambas drogas poseen un perfil farmacocinético similar.
